# Quantitative assessment of iron in heart and liver phantoms using dual-energy computed tomography

**DOI:** 10.3892/etm.2014.1813

**Published:** 2014-06-27

**Authors:** YI-SHAN TSAI, JIANG-SHIUH CHEN, CHIEN-KUO WANG, CHIA-HSING LU, CHAO-NENG CHENG, CHIN-SHUN KUO, YI-SHENG LIU, HONG-MING TSAI

**Affiliations:** 1 Department of Diagnostic Radiology, National Cheng-Kung University College of Medicine and Hospital, Tainan 704, Taiwan, R.O.C.; 2 Department of Pediatrics, National Cheng-Kung University College of Medicine and Hospital, Tainan 704, Taiwan, R.O.C.

**Keywords:** thalassemia, iron, dual-energy computed tomography, magnetic resonance imaging, Hounsfield units

## Abstract

The aim of the present study was to determine the correlation between dual-energy computed tomography (DECT) Hounsfield units (HU) and iron concentration, as well as the correlation between HU and magnetic resonance imaging (MRI)-derived R2^*^ values, in phantoms of the heart and liver tissue. Phantoms were constructed containing pig heart or liver tissue and varying concentrations of iron (0.1, 5, 10, 15, 20 and 25 mg/ml). The phantoms were then examined by DECT and MRI. Linear regression analysis was used to determine the correlations between HU and iron concentration and HU and R2^*^ values. The HU value of DECT increased with increasing iron concentrations in the liver and heart phantoms in a linear manner. The slope of the HU value change against iron concentration revealed that ΔH_80–140_ provided a better discernment of iron concentration as compared with ΔH_100–140_. The derived R^2^ values were all >0.9 for the associations of DECT and MRI measurements with iron concentrations. Therefore, DECT may be used for the determination of iron concentration in the liver and heart tissue, with the results correlating with those obtained with MRI.

## Introduction

Patients with thalassemia require long-term blood transfusions, thus, are often subject to iron accumulation and subsequent organ fibrosis (1). The effect of iron accumulation varies with the specific organ affected (2), and accumulation in the heart and liver is associated with significant clinical consequences (1,3). Knowledge of the degree of iron accumulation at an early stage allows for treatment planning, which may delay the progression of the condition (4). Typically, a definitive diagnosis of iron accumulation requires a biopsy, which is associated with the risks of an invasive procedure, including bleeding and infection. Thus, noninvasive diagnostic methods for determining the degree of iron accumulation have become a key area of research.

Magnetic resonance imaging (MRI) has been demonstrated to be useful for the evaluation of cardiac and hepatic iron accumulation (5). Cardiovascular MRI can provide data on cardiac muscle function, while the T2^*^ value obtained from multi-echo gradient recalled echo techniques indicates the degree of iron accumulation (6–8). However, despite MRI being a noninvasive test, it also has limitations. Cardiovascular and liver imaging examinations require the examinee to hold their breath to ensure a better image quality and a more accurate T2^*^ value. As a result, high quality images and results are difficult to obtain for certain patients, including young children and others who have difficulty holding their breath.

Research into dual-energy computed tomography (DECT) began as early as 1977, and the technology has been applied to liver hemochromatosis since the 1980s (9). Beginning in 1991, DECT has been used for the diagnosis of hepatic diseases (10). With continued advances in the technology, DECT has been applied for imaging of abdominal organs, the musculoskeletal and vascular systems, as well as for specific conditions, such as lithiasis and calcifications (11–14). The use of DECT in the assessment of iron accumulation in thalassemia patients was initially investigated in 1988 (15). Previous studies have indicated a correlation between DECT and MRI data used to detect iron accumulation in various organs (8,9).

In the present study, phantoms containing minced pig heart or liver and varying iron concentrations were examined with DECT and MRI. The aim of the study was to determine the correlation between CT Hounsfield units (HU) and iron concentration, as well as the correlation between HU and MRI-derived R2^*^ values.

## Materials and methods

### General information

The primary aim of the study was to mimic the heart and liver, the organs most often subject to transfusion-induced iron accumulation in thalassemia patients, and to use DECT to accurately access the quantity of accumulated iron. Therefore, fresh pig heart and liver specimens were obtained from a local slaughterhouse and specimens similar in size were selected for the study to emulate the internal environment of the human body. The models and methods used in the study have been validated in a number of previous studies (6–8). The study was approved by the Instititional Review Board of the National Cheng Kung University Hospital.

### Phantoms

Prior to preparing the phantoms, iron solution [50 mg/ml iron (III)-hydroxide polymaltose complex; Vifor (International), Ltd., St Gallen, Switzerland] was mixed with 0.9% NaCl solution (Sigma-Aldrich, St. Louis, MO, USA) to further prepare the solutions of the desired concentrations (0.1, 5, 10, 15, 20 and 25 mg/ml).

Iron solutions of various concentrations were added to test tubes that had been prefilled with minced pig heart or minced pig liver to prepare mud-like phantoms. One part iron solution was mixed with nine parts minced liver tissues or minced heart tissue (volume/volume) to achieve the final phantoms. In total, 34 four iron diluted solutions with minced heart or minced liver, plus one background tube with only normal saline, were prepared as sub-groups of the phantoms, and all were sealed to prevent oxidation. Next, four trays were prepared; two with normal saline, one with minced pig liver and one with minced pig heart. The test tubes containing minced pig liver and heart with varying concentrations of iron were then placed in the trays. Tubes with varying concentrations of iron and minced pig liver were placed in a normal saline tray and the tray containing minced pig liver, and tubes with varying concentrations of iron and minced pig heart were placed in a normal saline tray and the tray containing minced pig heart. Each corresponding tray had 35 test tubes as aforementioned. Every four trays were assigned as a set.

Each of the phantoms, including 10 sets (40 trays), were then examined with MRI and DECT. Three radiologists (A, B and C) separately performed the scanning of 10 sets of phantoms each for 10 times. The intra- and inter-observer reliability coefficients were determined. Generally, CT or MR values are determined by one time measurement. However, due to the signal-to-noise association, greater variation of the measurements is likely to occur at different signal intensities. For example, in CT imaging the signal-to-noise ratio is higher at 80 kVp than at 140 kVp; thus, greater variation in measurements occurs at 80 kVp. Determination of intra-class reliability was required for this reason, while the determination of inter-class reliability was necessary to examine the variation caused by different operators.

### MRI and DECT

Grade-echo MRI was performed on the phantoms with a Philips Achieva 1.5T A series MRI system (Philips Medical Systems, Andover, MA, USA) with the following settings: Coil, XL Torso (8 channels); parameters, fast field echo; repetition time, shortest; echo time, 2.3 (echospacing 2.3) with a total of eight echos; and flip angle, 20. The T2^*^ value was obtained, from which the R2^*^ value was calculated (R2^*^ = 1,000/T2^*^).

Single-energy computed tomography (SECT) imaging was performed with a Siemens SOMATOM Definition Flash system (Siemens Healthcare, Malvern, PA, USA) using the following settings: Voltage, 120 kVp; and tube current time, 200 mAseff. DECT with a tin filter was performed with the following settings: Pair voltage, 80/140 kVp; tube current time, 333×142 mAseff; computed tomography dose index (CTDI)_vol_, 13.2 mGy; pair two voltage, 100/140 kVp; tube current time, 333×283 mAseff; and CTDI_vol_, 27.1 mGy. Data were obtained at 80 and 140 kVp and the ΔH_80–140_ was calculated as the difference between the values at 80 and 140 kVp. Similarly, data were obtained at 100 and 140 kVp and the ΔH_100–140_ was calculated as the difference between the values at 100 and 140 kVp.

### Statistical analysis

Intra- and inter-class reliability were presented as the intra-class correlation coefficient (ICC) and κ values for the R2^*^ MRI measurement, respectively. The ICC value was defined as follows: >0.75, excellent; 0.59–0.75, good; 0.40–0.58, fair; and ≤40, poor (16), while the κ value was defined as follows: ≤0, poor; 0.01–0.20, slight; 0.21–0.40, fair; 0.41–0.60, moderate; 0.61–0.80, substantial; and 0.81–1, almost perfect (17). Higher ICC and κ values indicated greater reliability and less variation between the measurements. DECT and MRI R2^*^ data are represented as the mean ± standard deviation. Differences between conditions (normal saline and liver or heart) were compared using the two-sample t-test. A simple linear regression line was applied to identify the associations between DECT and MRI measurements with iron concentrations. Scatter plots with a predicted regression line were constructed comparing the HU measurements and iron concentration. Predicted regression lines were represented as y = b_1_x + b_0_, and corresponding R^2^ values were calculated. All the statistical assessments were two-tailed, and a value of P<0.05 was considered to indicate a statistically significant difference. Statistical analyses were performed using SPSS 18.0 statistical software (SPSS, Inc., Chicago, IL, USA).

## Results

### Intra- and inter-observer reliability of MRI

MRI R2^*^ data were used for determining the intra- and inter-observer reliability. For the heart phantom, the intra-observer reliability (ICC) was 0.986 for physician A, 0.981 for physician B and 0.98 for physician C. The inter-observer reliability coefficient (κ) was 0.918 between physician A and B, 0.928 between physician A and C and 0.923 between physician B and C. For the liver phantom, the intra-observer reliability (ICC) was 0.982 for physician A, 0.985 for physician B and 0.984 for physician C. The inter-observer reliability coefficient (κ) was 0.886 between physician A and B, 0.882 between physician A and C and 0.889 between physician B and C (data not shown). These results indicate that the intra- and inter-class reliability values were high.

### DECT and MRI R2^*^ measurements

Table I summarizes an representation of the DECT and MRI R2^*^ measurements for each of the four groups (liver and heart phantoms in normal saline and liver and heart phantoms in liver and heart, respectively), which were the average values from pooling the readings of the 34 tubes of each corresponding phantom group. The DECT and MRI measurements were consistent between the normal saline and minced heart and liver trays.

**Figure 1:**
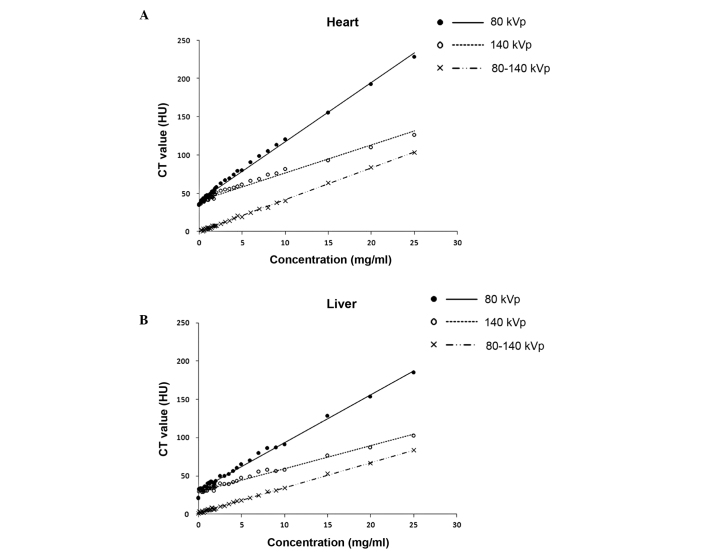
Scatter plots showing the predicted regression lines of DECT 80 kVp, 140 kVp and ΔH_80–140_ with iron concentrations in the (A) heart and (B) liver. DECT, dual-energy computed tomography.

Fig. 1 shows the DECT 80 and 140 kVp data compared with the iron concentrations for the heart and liver phantoms. In the heart, an increase in 1 mg/ml iron corresponded to an increase in CT HU values of 7.751 for DECT 80 kVp, 3.654 for 140 kVp and 4.066 for ΔH_80–140_. In the liver, an increase of 1 mg/ml iron corresponded to an increase in CT HU values of 6.265 for DECT 80 kVp, 3.010 for 140 kVp and 3.296 for ΔH_80–140_.

**Figure 2:**
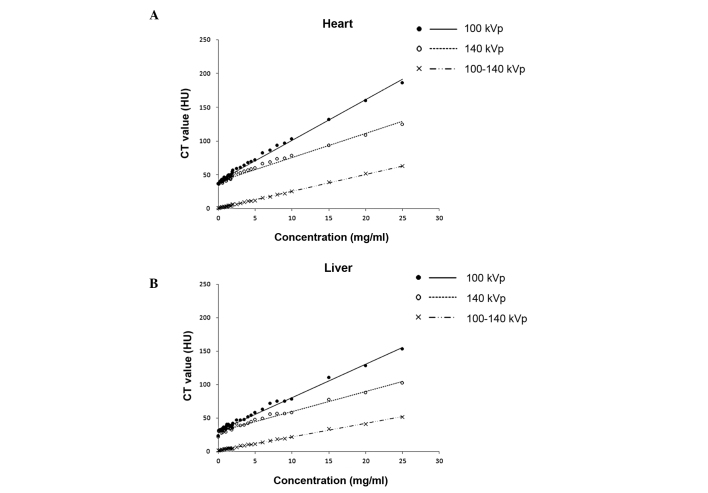
Scatter plots showing the predicted regression lines of DECT 100 kVp, 140 kVp and ΔH_100–140_ with iron concentrations in the (A) heart and (B) liver. DECT, dual-energy computed tomography.

Fig. 2 shows the DECT 100 and 140 kVp data compared with the iron concentrations for the heart and liver phantoms. In the heart, an increase of 1 mg/ml iron corresponded to an increase in CT HU values of 6.04 for DECT 100 kVp, 3.586 for 140 kVp and 2.505 for ΔH_100–140_. In the liver, an increase of 1 mg/ml iron corresponded to an increase in CT HU values of 4.996 for DECT 100 kVp, 3.018 for 140 kVp and 2.020 for ΔH_100–140_.

**Figure 3:**
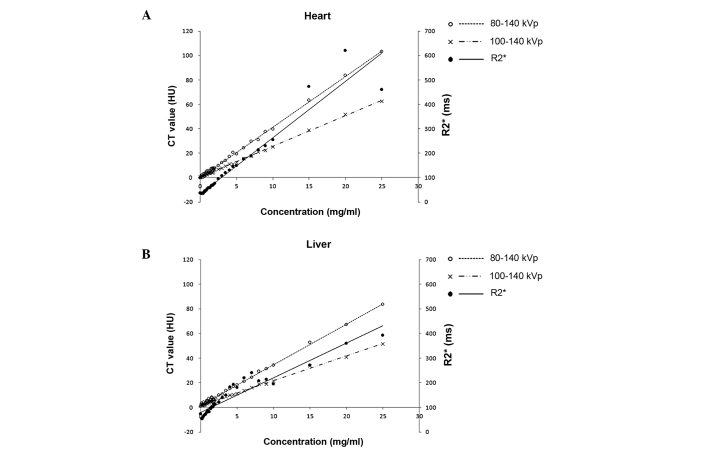
Scatter plots showing the predicted regression lines of DECT ΔH_80–140_, ΔH_100–140_ and R2^*^ with iron concentrations in the (A) heart and (B) liver. DECT, dual-energy computed tomography.

Scatter plots with predicted regression lines of DECT 80–140 kVp, DECT 100–140 kVp and MRI R2^*^ compared with iron concentrations are shown in Fig. 3. In the heart, an increase of 1 mg/ml iron corresponded to an increase in CT HU values of 4.147 for DECT 80–140 kVp and 2.505 for DECT 100–140 kVp, with the corresponding increase of 23.072 in the MRI R2^*^ value. In the liver phantoms, an increase of 1 mg/ml iron corresponded to an increase in CT HU values of 3.306 for DECT 80–140 kVp and 2.020 for DECT 100–140 kVp, with the corresponding increase of 14.04 in the MRI R2^*^ value.

Table II summarizes the predicted regression lines and corresponding R^2^ values for the associations between DECT and MRI measurements with iron concentrations. The derived R^2^ values were all >0.9, indicating that DECT and MRI measurements were significantly correlated in all the models.

## Discussion

The results of the present study using phantoms indicate that DECT may be useful for the determination of iron concentration in heart and liver tissues. The HU value of DECT increased with increasing iron concentrations in the phantoms in a linear manner, and values were significantly correlated with MRI R2^*^ data. The R^2^ value from the linear regression analysis for each individual technique revealed the goodness of fit for the model, and indicated that DECT correlated with MRI for iron quantification, although the correlation was not completely linear at high iron concentrations. Furthermore, the slope of the HU value change compared with the iron concentration demonstrated that ΔH_80–140_ provided a better discernment of iron concentration compared with ΔH_100–140_. As shown in Fig. 3, the slope was steeper at 80–140 kVp than at 100–140 kVp for the heart and liver; a steeper slope indicates a greater change in CT HU per unit of iron concentration, indicating a greater discriminatory power.

MRI has become a useful tool for the determination of iron content in various tissues (18). MRI scans can be produced with various echo times to alter the contrast between different organs. As echo times increase, the image of a particular organ darkens; however, when iron is present, the image of the organ darkens more rapidly. The time for an organ to become twice as dark is designated as the T2^*^ value, and T2^*^ is inversely proportional to the iron concentration (18). R2^*^, defined as 1,000/T2^*^, is directly proportional to the iron concentration (14). A number of studies have examined the use of MRI in the determination of cardiac and hepatic iron accumulation. In an animal study, Wood *et al* (19) demonstrated that MRI measurements of cardiac T2^*^ may be used for quantifying cardiac iron accumulation. Ghugre *et al* (20) studied 31 patients with transfusion dependent sickle cell disease and 48 patients with thalassemia major and accurately determined cardiac R2^*^ measurements. In addition, Carpenter *et al* (21) reported that R2^*^ values were significantly correlated with cardiac iron concentration. Determination of hepatic iron concentration by MRI has been studied more extensively than that of cardiac iron determination (5,18). Wood *et al* (22) evaluated the use of R2 and R2^*^ values for the determination of hepatic iron concentration in 102 patients with iron overload and 13 controls, and reported that R2 and R2^*^ values can accurately measure hepatic iron concentration.

While CT can detect increased tissue iron levels, quantification is not possible due to variations in CT attenuation. In addition, coexisting fat in the liver can affect CT attenuation, thus, the detection of iron. Wood *et al* (23) examined the use of quantitative CT (QCT) for the determination of liver iron concentration by comparing liver attenuation by QCT with MRI estimates of liver iron concentration in 37 patients with siderosis secondary to transfusions. The authors found that although the CT HU values correlated with the MRI data when the liver iron concentration was above the normal range, when the liver iron concentration was <8 mg/g dry weight of liver, quantitation was unable to be performed due to the variability in intrinsic liver attenuation.

In DECT, two energy settings are used simultaneously. This allows for the differentiation of materials based on their energy-associated attenuation characteristics, such as density (24). In a study using phantoms and DECT, Fischer *et al* (7) reported a significant linear correlation between liver iron concentration and HU. In addition, Joe *et al* (8) used DECT to analyze the iron concentrations in liver phantoms and in liver transplant candidates, and compared the results with those of MRI. In the phantom study of Joe *et al* (8), CT HU values were shown to be strongly correlated with iron concentration, as was the ΔH between 80 and 140 kVp. In patients with clinically important hepatic iron accumulation, DECT exhibited a similar diagnostic performance as MRI, with areas under the receiver operating characteristic curves of 0.881 and 0.897, respectively. Fewer studies have investigated the use of DECT in the determination of cardiac iron accumulation. Hazirolan *et al* (9) compared the results of DECT and cardiac MRI for the detection of myocardial iron in 19 patients with thalassemia and found that the HU values of septal muscle were strongly correlated with T2^*^ values, whereas no correlation was observed in the paraspinal muscle.

The primary limitation of the present study was the use of phantoms. While phantoms can emulate the characteristics of organs, the use of whole organs with varying iron concentrations, as determined by analytical methods, is preferable. However, this type of analysis is very expensive. An additional limitation is that the results obtained with phantoms may not be the same as when the technique is applied to living tissue. Finally, the fat content of the liver can affect DECT results and this was not assessed in the current study.

In conclusion, the results of the present study demonstrate that DECT can be used for the determination of iron concentration in liver and heart tissue, and that the results correlate with those obtained with MRI. However, further study is required on the use of DECT for the detection of heart and liver iron concentrations in humans.

## Figures and Tables

**Table I:** A representation of DECT and MRI R2^*^ measurements.

	Heart		Liver	
		
Group	Normal saline	Minced heart	Normal saline	Minced liver
DECT, HU				
80 kVp	71.20±44.15	71.44±44.36	55.63±35.93	56.61±35.9
140 kVp	53.73±21.22	54.74±21.09	39.7±17.38	41.62±17.38
ΔH_80–140_	17.67±23.36	16.90±23.71	16.11±18.88	15.16±18.85
DECT, HU				
100 kVp	64.74±35.71	65.03±34.61	50.29±29.43	51.3±28.66
140 kVp	53.26±20.72	54.44±20.67	40.08±17.97	41.74±17.41
ΔH_100–140_	11.68±5.35	10.80±14.31	10.38±11.80	9.73±11.55
MRI, msec				
R2^*^	114.82±121.60	127.35±136.95	156.63±92.39	137.36±83.91

DECT and MRI R2^*^ measurements for each of the four groups (liver and heart phantoms in normal saline and liver and heart phantoms in liver and heart, respectively) were the average values from the pooling of 34 tube readings from each corresponding phantom group. Data are presented as the mean ± standard deviation. R2^*^ = 1,000/T2^*^. No significant differences were observed between the normal saline and minced heart or the normal saline and liver groups. DECT, dual-energy computed tomography; MRI, magnetic resonance imaging; HU, Hounsfield unit.

**Table II:** Predicted regression lines and corresponding R^2^ values were used to identify the association between DECT and MRI measurements with iron concentrations.

	Heart		Liver	
		
Group	Predicted regression line	R^2^	Predicted regression line	R^2^
DECT, HU				
80 kVp	y = 7.75x + 39.6	0.996	y = 6.275x + 30.92	0.993
140 kVp	y = 3.65x + 39.76	0.980	y = 3.01x + 29.27	0.979
ΔH_80–140_	y = 4.15x − 0.10	0.998	y = 3.31x + 1.66	0.998
DECT, HU				
100 kVp	y = 6.04x + 40.27	0.993	y = 4.99x + 30.81	0.992
140 kVp	y = 3.59x + 39.74	0.982	y = 3.02x + 29.36	0.980
ΔH_100–140_	y = 2.51x + 0.53	0.999	y = 2.02x + 1.45	0.997
MRI, msec				
R2^*^	y = 23.07x + 32.75	0.926	y = 14.04x + 79.79	0.913

Predicted regression lines are represented as y = b_1_x + b_0_ and the corresponding R^2^ values were derived from simple linear regression analysis. DECT, dual-energy computed tomography; MRI, magnetic resonance imaging; HU, Hounsfield unit.
